# FKBP5 haplotypes and PTSD modulate the resting-state brain activity in Han Chinese adults who lost their only child

**DOI:** 10.1038/s41398-020-0770-5

**Published:** 2020-03-13

**Authors:** Rongfeng Qi, Yifeng Luo, Li Zhang, Yifei Weng, Wesley Surento, Neda Jahanshad, Qiang Xu, Yan Yin, Lingjiang Li, Zhihong Cao, Paul M. Thompson, Guang Ming Lu

**Affiliations:** 1 Department of Medical Imaging, Jinling Hospital, Medical School of Nanjing University, 210002 Nanjing, Jiangsu China; 2 Imaging Genetics Center, Mark and Mary Stevens Neuroimaging and Informatics Institute, University of Southern California, Marina del Rey, CA 90292 USA; 3 Department of Radiology, The Affiliated Yixing Hospital of Jiangsu University, 75 Tongzhenguan Road, 214200 Wuxi, China; 4 Mental Health Institute of the Second Xiangya Hospital, Central South University, China National Clinical Research Center for Mental Health Disorders, National Technology Institute of Psychiatry, 139 Middle Renmin Road, 410011 Changsha, Hunan China; 5 Psychology Department, Hangzhou Seventh People’s Hospital, 310013 Hangzhou, Zhejiang China

**Keywords:** Psychiatric disorders, Neuroscience

## Abstract

The stress-related gene *FKBP5* has been related to dysregulated glucocorticoid receptor (GR) signaling, showing increased GR sensitivity in trauma-exposed subjects with post-traumatic stress disorder (PTSD) but not in those without PTSD. However, the neural mechanism underlying the effects of *FKBP5* remains poorly understood. Two hundred and thirty-seven Han Chinese adults who had lost their only child were included. Four *FKBP5* single nucleotide polymorphisms (rs3800373, rs9296158, rs1360780, and rs9470080) were genotyped. All 179 participants were successfully divided into three *FKBP5* diplotype subgroups according to two major *FKBP5* H1 and H2 yin yang haplotypes. Brain average spectral power was compared using a two-way (PTSD diagnosis and *FKBP5* diplotypes) analysis of covariance within four separate frequency bands (slow-5, slow-4, slow-3, and slow-2). Adults with PTSD showed lower spectral power in bilateral parietal lobules in slow-4 and in left inferior frontal gyrus (IFG) in slow-5. There was significant *FKBP5* diplotype main effect in anterior cingulate cortex (ACC) in slow-4 (H1/H1 higher than other two subgroups), and in precentral/postcentral gyri and middle cingulate cortex (MCC) in slow-3 (H2/H2 higher than other two subgroups). Also, there was a significant diagnosis × *FKBP5* diplotype interaction effect in right parietal lobule in slow-3. These findings suggest that adults with PTSD have lower low-frequency power in executive control network regions. Lower power in ACC and greater power in the motor/sensory areas in *FKBP5* high-risk diplotype group suggest a disturbance of emotional processing and hypervigilance/sensitization to threatening stimuli. The interaction effect of diagnosis × *FKBP5* in parietal lobule may contribute to PTSD development.

## Introduction

Post-traumatic stress disorder (PTSD) is characterized by dysregulation of the hypothalamus-pituitary-adrenal (HPA) axis after exposure to traumatic events, displayed as enhanced negative-feedback inhibition of the HPA axis^1,2^. One typical clinical feature of this abnormality in PTSD is an exaggerated suppression of cortisol in response to dexamethasone administration and the enhanced sensitivity of glucocorticoid receptors (GR)^3,4^. Even so, only a minority of trauma-exposed individuals will eventually develop PTSD^5^, and the increased GR sensitivity following stress happens only in PTSD but not in non-PTSD subjects^3,4^. The underlying mechanisms of the association between PTSD development and GR sensitivity remain poorly understood.

Genetic factors that regulate GR signaling influence the individual differences in the HPA axis response to stress^6,7^. A critical regulatory gene is the FK506-binding protein 5 (*FKBP5*) gene which encodes the FKBP5 protein^8^. In the absence of cortisol, FKBP5 binds to the GR complex, resulting in decreased affinity for cortisol and less efficient nuclear translocation of GR. In the presence of cortisol, FKBP5 is exchanged with other co-chaperones, causing more efficient translocation of GR to the nucleus^9^. Healthy carriers of specific *FKBP5* single nucleotide polymorphisms (SNPs) were tended to have higher FKBP5 protein expression and thus decreased cortisol affinity and nuclear translocation of GR, resulting in GR resistance^6^, suggesting the presence of protective features after response to stress. However, this functional association is switched in PTSD^6^, in which the same *FKBP5* SNPs were associated with increased GR sensitivity, conferring “risk” SNPs for PTSD. So far, little is known about the exact mechanisms of different modulatory effects of *FKBP5* risk SNPs in people who experienced traumatic events with and without PTSD.

Imaging genomics is significantly improving our understanding of human brain function, and offers a means to study molecular and cellular abnormalities that are implicated in many psychiatric disorders in which genes play an important role^10–13^. To the best of our knowledge, only one imaging genomics study with a small sample size (a total of 54 subjects) has investigated the effect of *FKBP5* risk SNP polymorphisms on brain function of PTSD^14^. In that study, Fani et al. firstly reported that PTSD patients who carried two *FKBP5* rs1360780 risk SNPs had lower fractional anisotropy in the cingulum, compared to other diagnostic and genotype groups. Also, trauma-exposed subjects who carried two *FKBP5* rs1360780 risk SNPs had lower functional connectivity of hippocampus-anterior cingulate cortex than non-risk SNP carriers, regardless of PTSD diagnosis^14^. The *FKBP5* rs1360780 has been demonstrated to function together with other *FKBP5* SNPs which are in strong linkage disequilibrium, resulting in gene dose-dependent regulation on the HPA axis^6^. Several studies have focused on the two major functional *FKBP5* haplotypes driven from four SNPs (rs3800373, rs9296158, rs1360780, and rs9470080)—the H1 (carrying zero risk SNPs: AGCC) and H2 (carrying four risk SNPs: CATT) yin yang haplotypes^15,16^. However, no imaging genomics study to date has used this desirable haplotype to investigate the modulatory effects of *FKBP5* on brain function in PTSD development. In a recent fMRI study examining effects of *FKBP5* risk SNPs, Bryant et al. divided the healthy participants into high-risk and low-risk genogroups based on the number of risk alleles carried (using the above-mentioned four *FKBP5* SNPs) and then investigated the differences in resting-state brain function between these two genogroups^17^. They found that the high-risk genogroup showed less power in low frequency range but more power in higher frequency range in the frontotemporoparietal network, compared to the low-risk genogroup.

In this prospective study, we aimed to examine the modulatory effects of *FKBP5* H1 and H2 yin yang haplotypes on resting state brain function using resting-state fMRI in trauma-exposed subjects with and without PTSD. A wide variation in the types of trauma and differences in race/ethnicity of the populations studied might potentially influence the findings of imaging genomics analyses^18^. Therefore, we conducted a PTSD survey in a specific group of Han Chinese adults who had lost their only child. By doing so, we restricted our study to participants of the same ethnicity (Han Chinese) and homogenous traumatic event (loss of only child). Power spectrum analysis^19,20^—an important fMRI approach that measures the strength of intrinsic brain activity—was used in this study for three reasons. Firstly, the power spectrum, especially within the low-frequency band, is a physiologically meaningful and powerful biomarker in exploring human spontaneous brain activity^20–22^, and has been successfully applied in prior genetic association studies in several mental and neuropsychiatric disorders such as mild cognitive impairment^23,24^ and major depressive disorder^25^. Secondly, in a prior imaging genomics study using four *FKBP5* risk SNPs, only spectral power demonstrated significant differences between the high-risk and low-risk genogroups^17^. Thirdly, the power spectrum analysis is a whole-brain algorithm that needs no region-specific hypothesis, which might be more suitable for a preliminary study. We hypothesized that the *FKBP5* haplotypes would exhibit different modulatory effects on brain spectral power in Han Chinese adults who had lost their only child with and without PTSD.

## Methods

### Subjects

The present study was approved by the Medical Research Ethics Committee of Jiangsu University. Each participant provided written informed consent. Between September 2016 and March 2017, we conducted a survey in Jiangsu Province, China on a population of the Han Chinese adults who had lost their only child during the period of “One-Child Policy”^26,27^. All 237 Han adults who had lost their only child—without other major traumatic exposures based on the clinician-administered PTSD scale (CAPS) life events checklist—were successfully interviewed and screened by the clinician-administered PTSD scale (CAPS). They were also further screened with the Chinese version of the structured clinical interview for DSM-IV (SCID)^28^ which was revised by Prof. Lipeng Fei from the Beijing Hui Long Guan Hospital. After doing this, 170 out of the total of 237 adults did not meet any diagnostic criteria for mental illness (including current or lifetime PTSD) or substance use disorders; 57 adults were diagnosed with PTSD; the remaining 10 adults were diagnosed with other psychiatric disorders (5 with major depressive disorder, 4 with generalized anxiety disorder, and 1 with both depression and anxiety diagnosis) and they were not included in the current study.

Exclusion criteria for the following MRI study were as follows: any current or history of brain injury or other major medical or neurological conditions (4 adults without PTSD were excluded for cerebral infarction or ischemia, and 1 adult without PTSD was ruled out for a history of major depressive disorder and antidepressant drug therapy); any MRI contraindication (none); and left-handedness (none).

### MR scanning

MR imaging was performed with a 3-T scanner (Achieva 3.0 TTX; Philips, Amsterdam, the Netherlands). Each participant was instructed to stay still in the scanner during image acquisition, keep eyes closed, remain awake and move as little as possible. T_1_-weighted anatomical images were acquired using a three-dimensional turbo fast echo sequence (repetition time ms/echo time msec [TR/TE], 9.7/4.6; flip angle, 9°; field of view (FOV), 256 × 256 mm^2^; matrix size, 256 × 256; slice thickness, 1 mm; 160 sagittal slices). For resting-state functional imaging, we performed a single-shot, gradient-recalled echo-planar imaging sequence (TR/TE, 2000/30; flip angle, 90°; FOV = 192 × 192 mm^2^; matrix, 64 × 64; voxel size, 3 × 3 × 4 mm^3^; volume number, 230; 35 axial slices). The total scan time for resting-state functional imaging was 460 s.

### Measures

All participants were assessed with neuropsychological tests, which included: the Hamilton Depression (HAMD)^29^ and Hamilton Anxiety (HAMA)^30^ rating scales, Mini-Mental State Examination (MMSE)^31^, Chinese Social Support Rating Scale (SSRS) with sections about subjective support, objective support and the utility of support^32^, and individual Simple Coping Style Questionnaire (SCSQ) with active and negative coping score, and the coping tendency score^33^. A detailed description is available in the online Supplementary Note 1.

### Data preprocessing

MRI data was preprocessed with the Data Processing Assistant for Resting-State fMRI (DPARSF, http://rfmri.org/DPARSF) which is based on Statistical Parametric Mapping (http://www.fil.ion.ucl.ac.uk/spm). First, the initial ten volumes were excluded. Then, the slice timing and head motion correction were conducted on all the remaining volumes. Individual T_1_-weighted images were co-registered to the functional images and then segmented into gray matter, white matter, and cerebrospinal fluid, and transformed into the standard Montreal Neurological Institute (MNI) space using the Diffeomorphic Anatomical Registration Through Exponentiated Lie algebra (DARTEL) method^34^. Finally, the functional images were transformed into the MNI stereotaxic space (3 × 3 × 3 mm^3^), using the parameters of the T_1_-weighted image normalization, and then smoothed with an 8 mm full width at half maximum (FWHM) isotropic Gaussian kernel.

### Quality control and nuisance regression

To minimize head motion confounds, we used the Friston 24-parameter model^35^ to regress out head motion effects. Individual head translations, rotations, and framewise displacement (using the Jenkinson formula) were calculated. Seven adults were excluded (5 without PTSD, and 2 with PTSD) for head translations > 1.5 mm or rotations > 1.5°, and 2 adults (without PTSD) were excluded for mean framewise displacement > 2.5 standard deviations. The mean framewise displacement of each participant was also included as a nuisance covariate in the statistical analysis of the fMRI data^36,37^. Mean signals from cerebrospinal fluid and white matter were also regressed out as spurious variance to restrict the analysis to gray matter.

After performing the quality control, a total of nine participants were excluded, and 55 adults with PTSD and 158 adults without PTSD remained.

### Power spectrum analysis

Firstly, for a given voxel, the time course was converted to the frequency domain using a fast Fourier transform^20,22^. Then, the square root of the power spectrum was computed and averaged across a predefined frequency interval. Based on the most current knowledge, some brain disorders^38,39^, as well as some genes^17^, may selectively affect the brain power spectrum within a certain frequency band. Here, we have divided the full frequency range (0–0.25 Hz) into four narrowly-defined bands according to prior studies^21,22^: slow-5 (0.01–0.027 Hz), slow-4 (0.027–0.073 Hz), slow-3 (0.073–0.198 Hz), and slow-2 (0.198–0.25 Hz); and we calculated the average power spectrum in individual frequency band. For standardization purposes, the average power spectrum of each voxel was divided by the global mean values.

### DNA genotyping

With the exception of three adults without PTSD who refused the blood collection procedure, we successfully collected DNA data from all other participants from peripheral blood samples. Four *FKBP5* SNPs (rs3800373, rs9296158, rs1360780, and rs9470080) were genotyped using the Improved Multiple Ligase Detection Reaction (iMLDR) technique developed by Genesky Biotechnologies, Inc. (Shanghai, China)^40^. Detailed primers for these four SNPs of *FKBP5* are listed in Supplementary Table 1. About 5% of the samples were randomly selected for confirmation, and the results were 100% concordant. For all 210 study participants with available gene data—and who passed quality control for their fMRI data—the distributions of rs3800373, rs9296158, rs1360780, and rs9470080 did not differ from Hardy-Weinberg equilibrium (*P* = 0.74, 0.45, 0.62, and 0.30, respectively) calculated using R, version 3.5.3 (https://www.r-project.org).

### *FKBP5* haplotype estimation

**Fig. 1:**
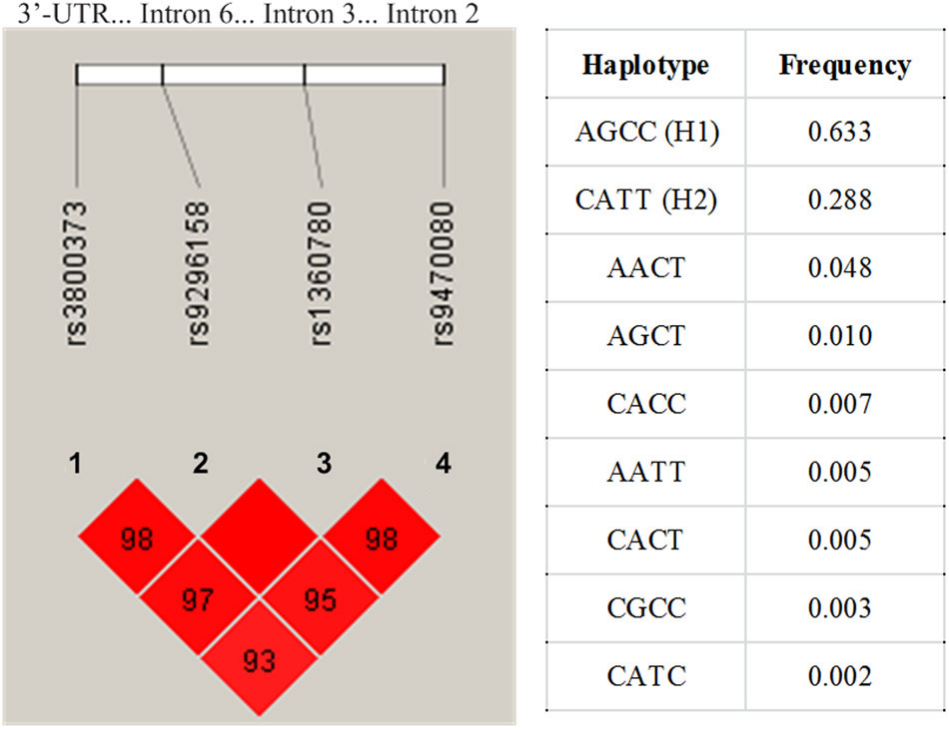
The *FKBP5* 4-SNP haplotype block structure, and two major H1 (yin) H2 (yang) functional haplotypes. The *FKBP5* H1 (yin) haplotype carries zero risk SNPs (AGCC); the H2 (yang) haplotype carries all four risk SNPs (CATT). H1 and H2 haplotypes account for 92.1% of the haplotype diversity in this study. SNP single-nucleotide polymorphism, UTR untranslated regions.

A haplotype is a combination of alleles that are located close together on the same chromosome and inherited together^41^. Here, we used Haploview version 4.2^42^ and genotype data to compute the linkage disequilibrium blocks for these four *FKBP5* SNPs, and found they were in strong linkage disequilibrium (Fig. 1) and in approximate allelic identity (Supplementary Table 2). There were 9 *FKBP5* haplotypes (frequency ≥ 0.01) identified in this study, with two major functional haplotypes—the H1 and H2 yin yang haplotypes (Fig. 1). The H1 (yin) haplotype carries zero risk SNPs (AGCC), while the H2 (yang) haplotype carries all four risk SNPs (CATT)^6,15,16^. These two major functional haplotypes accounted for 92.1% of haplotype diversity in this study, which is similar to findings in prior studies^15,16^. Then, PHASE version 2.1^43–45^ was performed to determine the most probable haplotype assignments for each individual by assessing the probability of each possible haplotype and determining a confidence score. A diplotype represents a pair of haplotypes on homologous chromosomes and could provide more complete genetic information^46,47^. For this reason, the *FKBP5* diplotype was further estimated for 210 study participants; 179 of them carried homozygote and heterozygote combinations of the H1 and H2 yin yang haplotypes and were included in the final diplotype based neuroimaging analysis. From these 179 adults, 87 were H1/H1 (64 without PTSD, 23 with PTSD), 72 were H1/H2 (51 without PTSD, 21 with PTSD), and 20 were H2/H2 (15 without PTSD, 5 with PTSD) (Supplementary Table 3).

### Statistical analysis

SPSS version 25 (IBM Corp, Armonk, New York, USA) was used to analyze the demographic and neuropsychological data. A two-way (diagnosis of PTSD, *FKBP5* diplotypes) analysis of variance was used to evaluate the main effects of diagnosis, *FKBP5* diplotypes and their interaction effects on demographic and neuropsychological data. A voxel-wise two-way (diagnosis, *FKBP5* diplotypes) full factorial analysis of covariance (ANCOVA) was performed using SPM12 to assess the main effect of PTSD diagnosis, *FKBP5* diplotypes and their interaction effects on brain power spectrum maps within the four different frequency bands, adjusting for effects of age, sex, educational level, duration since child-loss trauma, and framewise displacement due to head motion. The frequency band was not considered as a repeated factor in this paper as we wanted to primarily focus on the effects that diagnosis and genetic variation may exert on the power spectrum within different bands, but not the differences among different bands themselves. Results were corrected for multiple comparisons (corrected *P* < 0.05) using random-field theory (RFT)^48^ with initial voxel level *P* < 0.001 and cluster level *P* < 0.05.

A partial correlation analysis was performed to examine the relationship between regions with significantly different power spectra and CAPS, SSRS, SCSQ, HAMA, and HAMD, with the inclusion of age, sex, educational level, duration since child-loss trauma, and head motion as covariates. Correlation results were corrected for multiple comparisons using the Bonferroni correction for the number of regions where altered power spectra were detected from the two-way ANCOVA (cut-off *P* values of 0.05/8 = 0.006, corresponding to all eight regions showing differences in this study).

## Results

### Clinical and neuropsychological data

A flowchart describing the study population is detailed in Supplementary Fig. 1. All 179 adults (49 with PTSD and 130 without PTSD) who carried homozygote and heterozygote combinations of the H1 (yin) and H2 (yang) haplotypes were included in the final *FKBP5* diplotypes based neuroimaging analyses (Table 1 and Supplementary Table 3).

**Table 1:** Demographics and neuropsychological data of Han Chinese adults who lost and only child and carried heterozygous/homozygous combinations of 2 major *FKBP5* haplotypes.

Protocols	Adults with PTSD (*n* = 49)	Adults without PTSD (*n* = 130)	*P* value
Age (±SD) (years)	57.76 ± 5.59	58.79 ± 5.46	0.26^a^
Sex (F/M)	35/14	60/70	0.003^b^
Education (years)	6.43 ± 4.24	6.58 ± 3.58	0.82^a^
HAMD	15.84 ± 6.77	5.93 ± 4.19	<0.001^a^
HAMA	12.65 ± 6.71	4.57 ± 3.41	<0.001^a^
MMSE	25.69 ± 3.17	26.13 ± 3.44	0.44^a^
Duration since child-loss trauma, month	59.71 ± 49.82	107.09 ± 72.39	0.001^a^
CAPS_total	46.69 ± 12.66	16.35 ± 9.95	<0.001^a^
*SSRS*			
Objective support	12.27 ± 2.77	12.64 ± 2.75	0.42^a^
Subjective support	21.55 ± 3.82	21.47 ± 3.89	0.90^a^
Utility of support	5.63 ± 2.07	5.48 ± 1.94	0.65^a^
SSRS_total	39.45 ± 7.13	39.58 ± 6.64	0.91^a^
*SCSQ*			
Active	18.27 ± 6.35	19.38 ± 6.46	0.31^a^
Negative	10.08 ± 3.04	10.37 ± 3.34	0.60^a^
Copying tendency	8.18 ± 5.70	9.05 ± 6.01	0.38^a^

*PTSD* post-traumatic stress disorder, *HAMD* Hamilton depression, *HAMA* Hamilton anxiety, *MMSE* mini-mental state examination, *CAPS* clinician-administered PTSD scale, *SSRS* social support rating scale, *SCSQ* simple coping style questionnaire.

Values are expressed as mean ± SD.

^a^The *P* value for the difference between the two trauma-exposed groups was obtained by two-sample *t*-test.

^b^The *P* value for gender distribution between the two trauma-exposed groups was obtained by the chi-square test.

There were no significant differences between PTSD and non-PTSD groups in age, educational level, SSRS, or SCSQ (all *P* > 0.05), but the PTSD group showed higher CAPS, HAMA and HAMD scores, higher male/female ratio, and shorter duration since losing the child (Table 1) than the non-PTSD groups. There was no significant diagnosis or *FKBP5* diplotypes main effect, and no interaction effect on other clinical and neuropsychological data.

### The main effect of PTSD diagnosis

**Fig. 2:**
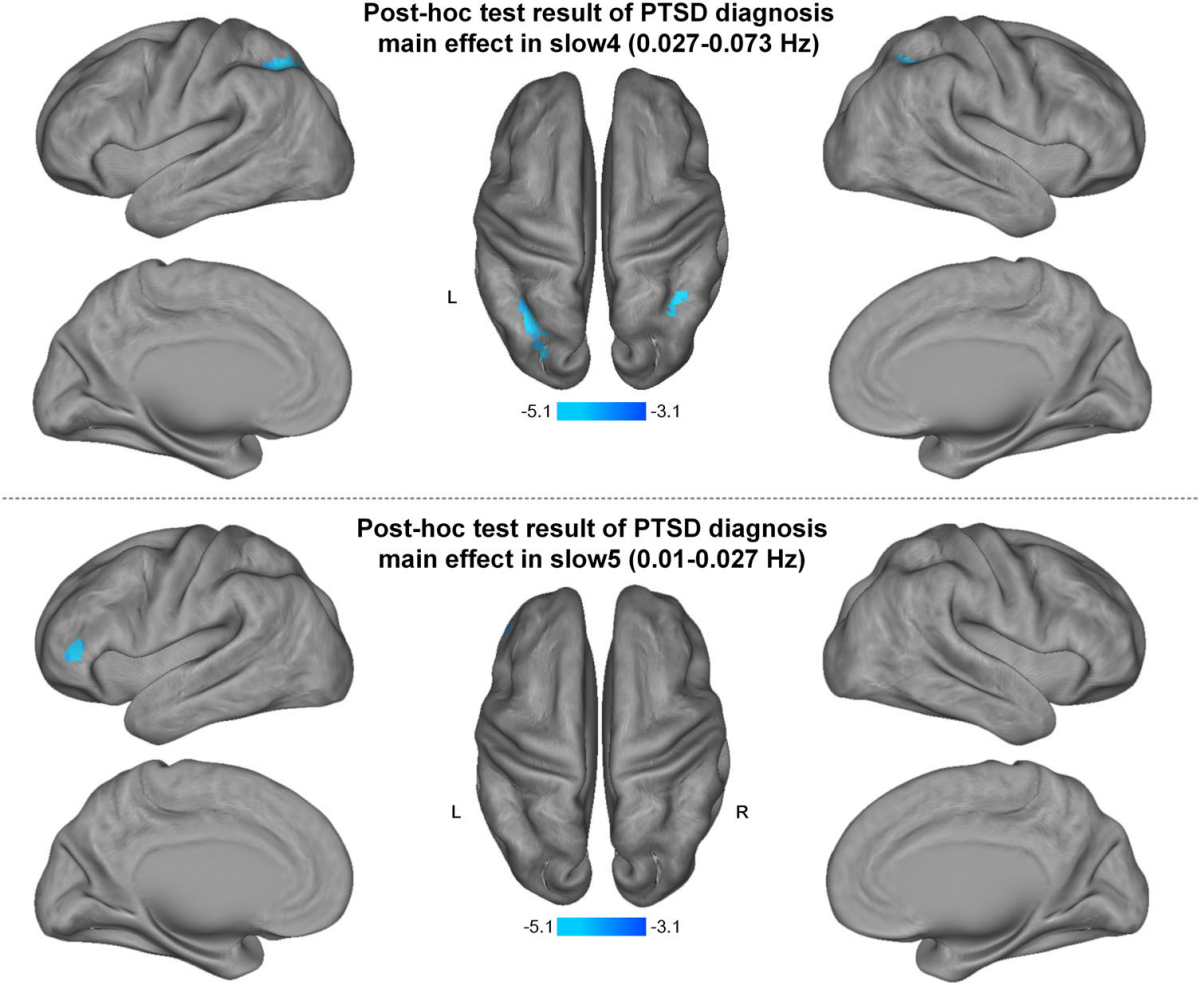
Post-hoc test result of PTSD diagnosis main effect on brain resting-state power spectrum (corrected *P* < 0.05). Post-hoc analysis of PTSD main effect shows that PTSD adults have lower spectral power in bilateral parietal lobules in slow-4 and in left inferior frontal gyrus in slow-5 band, relative to trauma-exposed adults without PTSD.

Significant PTSD diagnosis effects on spectral power were found in bilateral parietal lobules in slow-4 band and in left inferior frontal gyrus (IFG) in slow-5 band. Post-hoc analysis showed that PTSD adults had lower spectral power in these regions, relative to trauma-exposed adults without PTSD (Fig. 2 and Supplementary Table 4).

### The main effect of *FKBP5* diplotypes

**Fig. 3:**
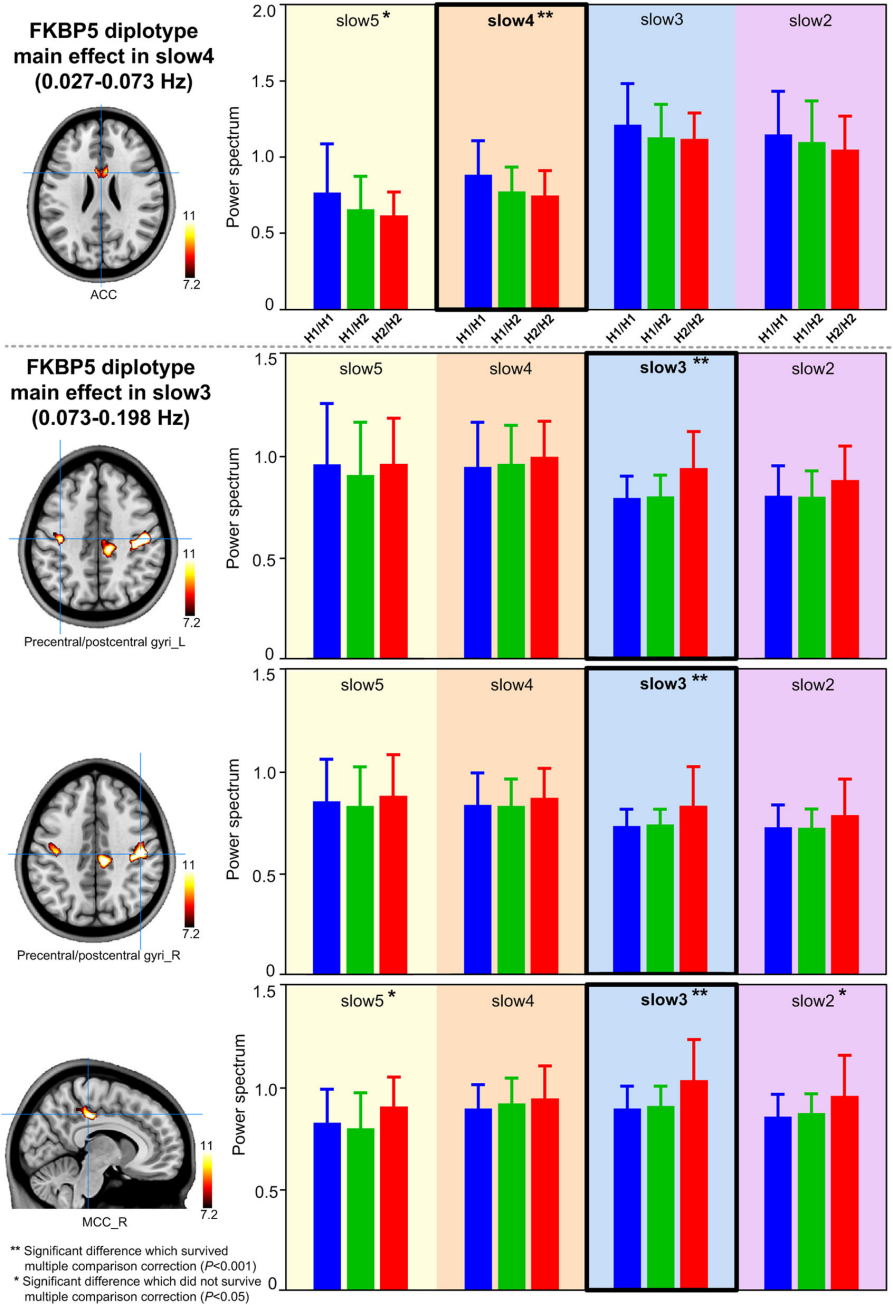
Main effects of *FKBP5* diplotypes on brain resting-state power spectrum (corrected *P* < 0.05). There is significant *FKBP5* diplotype main effect on spectral power in the anterior cingulate cortex (ACC) in the slow-4 band (H1/H1 higher than the other two genogroups), and in the bilateral precentral/postcentral gyri and middle cingulate cortex (MCC) in the slow-3 band (H2/H2 higher than the other two genogroups), irrespective of PTSD diagnosis.

There was significant *FKBP5* diplotypes main effect on spectral power in the anterior cingulate cortex (ACC) in slow-4 band, and in the bilateral precentral/postcentral gyri and middle cingulate cortex (MCC) in slow-3 band in all participants, irrespective of PTSD diagnosis (Fig. 3 and Supplementary Table 4). At ACC, the H1/H1 diplotype subgroup had higher spectral power than other two genogroups. For the precentral/postcentral gyri and MCC, the H2/H2 diplotype group had higher spectral power than other two genogroups. The *FKBP5* diplotype modulation accounted for 14% of the variance in spectral power in left precentral/postcentral gyri, 10% in right precentral/postcentral gyri, 10% in the right MCC and 9% in the ACC (partial eta squared [*η*^2^] = 14, 10, 10, and 9%, respectively).

### Interaction of *FKBP5* diplotypes and PTSD diagnosis

**Fig. 4:**
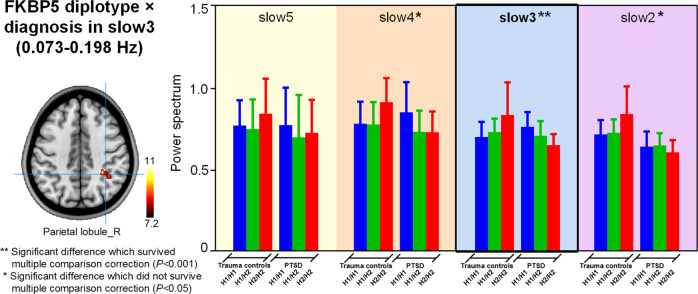
Interaction effect of diagnosis × *FKBP5* diplotypes effect on brain resting-state power spectrum (corrected *P* < 0.05). There is a significant diagnosis × *FKBP5* diplotype interaction effect on spectral power in right parietal lobule in slow-3 band. Within non-PTSD adults, the H2/H2 diplotype subgroup has higher spectral power than the other two genogroups; whereas within PTSD adults, the H2/H2 diplotype subgroup has lower spectral power than the other two genogroups.

There was a significant diagnosis × *FKBP5* diplotype interaction effect on spectral power in right parietal lobule in slow-3 band (Fig. 4 and Supplementary Table 4). Post-hoc analysis showed that within non-PTSD adults, the H2/H2 diplotype subgroup had higher spectral power than other two genogroups; whereas within PTSD adults, the H2/H2 diplotype subgroup had lower spectral power than the other two genogroups (Fig. 4). This diagnosis × FKBP5 diplotype interaction accounted for 10% of the variance in spectral power in right parietal lobule (*η*^2^ = 10%).

### Partial correlation results

A marginally negative partial correlation was found between the power spectrum in right parietal lobule in slow-4 band and CAPS scores, albeit only in the PTSD group (*r* = −0.27; *P* = 0.06, Supplementary Fig. 2). Power spectra in brain regions affected by *FKBP5* diplotypes and diagnosis × *FKBP5* interaction were not correlated with any clinical or neuropsychological indices.

### Additional analyses of structural data

To evaluate whether the functional results in this study might be confounded by brain structural changes, we further performed voxel-based morphometry (VBM) to examine possible structural deficits, using the CAT12 Toolbox (http://dbm.neuro.uni-jena.de/cat12/). We used the default settings detailed in the manual for CAT12 (http://dbm.neuro.uni-jena.de/cat12/CAT12-Manual.pdf) except for applying the affine regularization using the International Consortium for Brain Mapping template for East Asian brains. The individual T_1_-weighted images were finally segmented into gray matter, white matter and cerebrospinal fluid. The segmented gray matter images were smoothed with an FWHM of 8 mm. Regional gray matter volume of each region showing significant differences in *FKBP5* diplotypes based neuroimaging analyses was extracted and then compared with a similar two-way ANCOVA. No significant diagnosis or *FKBP5* main effect or their interaction effect on gray matter volume was found in these regions.

## Discussion

In this study, we investigated the effects of PTSD diagnosis and stress-related gene *FKBP5* on spontaneous brain activity in Han Chinese adults who had lost their only child. We found a main effect of PTSD diagnosis on low-frequency power (slow-4 and slow-5 bands) in the parietal lobules and inferior frontal gyrus, and a main effect of *FKBP5* diplotype on low-frequency power (slow-4 band) in ACC and middle-frequency power (slow-3 band) in the motor/sensory areas irrespective of PTSD diagnosis. Also, there was a significant diagnosis × *FKBP5* interaction effect on middle-frequency power (slow-3 band) in parietal lobule.

The prominent neuroanatomical theory of PTSD points to a decreased prefrontal inhibitory control over the amygdala^49^, supported by quantitative neuroimaging studies^50,51^ and meta-analytic reviews^49,52^. Other evidence also supports the cognitive-affective imbalance theory in the pathology of PTSD^53,54^, as evidenced by underactivated regions within the brain executive system and overactivated regions within the emotional processing system. The parietal lobule is a core component of the brain executive control network^55,56^. Decreased parietal lobule activity has been demonstrated in adults with a history of early life stress exposure^57,58^ and patients with PTSD^54,59^. Its activity has also been found to be negatively correlated with PTSD symptoms^54^. Both right^60^ and left^61^ inferior frontal gyri play an important role in attentional monitoring and inhibiting inappropriate responses. Reduced inferior frontal gyrus activity during a proactive inhibition task was reported in veterans with PTSD, relative to control veterans without PTSD^62^. Thus, in this study, the lower low-frequency power in parietal lobule and inferior frontal gyrus aligns with the findings in prior studies about PTSD. The marginally negative correlation between parietal lobule and CAPS in PTSD adults in this study also supports our findings.

Twin studies posit that PTSD is moderately heritable, with heritability estimates in the range of 28–46%^63–65^. Increased GR sensitivity is only found in trauma-exposed subjects with PTSD rather than those without PTSD^6^. As a critical modulator of GR sensitivity, *FKBP5* is thought to be an interesting candidate gene for PTSD development^66^. Individuals with PTSD who carried two *FKBP5* rs1360780 risk SNPs (TT) exhibited the lowest cingulum fractional anisotropy, compared to the same risk SNPs carriers without PTSD, and other genotypes both with and without PTSD^14^. The *FKBP5* diplotypes with homozygote and heterozygote combinations of the H1 and H2 yin yang haplotypes provide more complete genetic information^46,47^. A recent study divided a group of healthy participants into high and low-risk genogroups according to the number of risk alleles of four *FKBP5* SNPs, and found that the *FKBP5* high-risk allele group demonstrated lower low-frequency power spectrum but greater high-frequency power spectrum in brain frontotemporoparietal network, compared to the low-risk allele group^17^. To the best of our knowledge, our current study is the first to further describe the effect of *FKBP5* diplotypes on resting-state brain activity in trauma-exposed subjects both with and without PTSD diagnosis. In this study, at ACC, the zero-risk diplotype group (H1/H1) displayed higher low-frequency power spectrum than the middle (H1/H2)- and high (H2/H2)- risk diplotype groups; while at precentral/postcentral gyri and MCC, the H2/H2 diplotype group had higher middle spectral power than other two genogroups. Although using different study samples and different *FKBP5* gene grouping methods, both our current results and findings from prior study^17^ suggest that the *FKBP5* risk alleles may have different modulatory effects on brain activity within different frequency bands, where high-risk alleles may associate with lower low-frequency power but greater middle or high-frequency power in frontotemporoparietal regions. In the *FKBP5* high-risk genogroup, the lower power spectrum in ACC may indicate impaired emotional processing, while the higher power spectrum in precentral/postcentral gyri may suggest hypervigilance/sensitization to threatening stimuli^17^. This interpretation is partially supported by a prior study on *FKBP5* rs1360780 in subjects recruited from the general medical clinics, where risk allele (TC/TT) carriers showed attention bias toward the threat, compared to non-risk allele carriers (CC)^67^.

In the present study, another important finding is the interaction modulatory effect of *FKBP5* × diagnosis in the parietal lobule, where in PTSD adults, the high-risk diplotype group was associated with lower spectral power than the other two genogroups, whereas, in non-PTSD adults the opposite occurred. One possible interpretation for the different modulatory effects of *FKBP5* risk diplotype in PTSD and non-PTSD subjects is that some brain regions—such as the parietal lobule—may have a resilient or compensatory role in trauma-exposed subjects without PTSD. Further research is needed to gain a precise understanding of the role of *FKBP5* risk diplotype on PTSD development. For example, more quantitative neuroimaging-genetics studies are required to provide reliable and repeatable intermediate phenotypes that would improve our understanding of GR sensitivity differences in subjects with PTSD and without PTSD.

This study has several limitations. First, our study only focused on the influence of losing an only child in China, and so we urge caution when applying these results to other traumatic experiences and other populations of different race or ethnicity. Second, due to the cross-sectional nature of the current study, it was unclear if the brain differences were present before the traumatic experience or if they occurred after the traumatic event. Third, an increasing number of studies have demonstrated that the *FKBP5* risk SNPs often interact with individual childhood trauma, resulting in an increased risk of PTSD development and greater PTSD symptoms^66,68^. This evidence suggests the importance of gene × childhood environment interactions for PTSD. However, childhood trauma data was not originally collected in this study and thus needs to be taken into account in further studies. Finally, given the significant history of failure to replicate candidate-gene studies^69,70^, the findings here should be considered as preliminary results and need to be validated by studies involving large replication samples or using data from genome-wide association studies (GWAS) of PTSD.

## Conclusion

In conclusion, our findings suggest that PTSD may impair lower low-frequency power in executive control network regions in Han Chinese adults who had lost their only child. The *FKBP5* high-risk diplotype group displayed lower spectral power in ACC and greater power in the motor/sensory areas, suggesting a disturbance of emotional processing and hypervigilance/sensitization to threatening stimuli. The interaction effect of diagnosis × *FKBP5* in parietal lobule may contribute to PTSD development in adults who experienced the loss of an only child.

## Supplementary information

Supplementary Material
