# Supplementary material for: FKBP5 haplotypes and PTSD modulate the resting-state brain activity in Han Chinese adults who lost their only child
Source: Transl Psychiatry. 2020 Mar 13;10:91. doi: 10.1038/s41398-020-0770-5 (PMC7070023; doi:10.1038/s41398-020-0770-5)
Supplement: Supplementary file 1 — Supplementary Material [file 41398_2020_770_MOESM1_ESM.docx]

**Supplementary Material**

1. **Supplementary Note 1: Measures of Neuropsychological Tests**
2. **Supplementary Tables**
3. **Supplementary Figures**
4. **Supplementary Note 1: Measures of Neuropsychological Tests**

All participants were assessed with neuropsychological tests included: Hamilton Depression (HAMD)[^1^](#_ENREF_1) and Hamilton Anxiety (HAMA)[^2^](#_ENREF_2) rating scales, Mini-Mental State Examination (MMSE)[^3^](#_ENREF_3), Chinese Social Support Rating Scale (SSRS), containing subjective and objective support, and the utility of support[^4^](#_ENREF_4), and individual Simple Coping Style Questionnaire (SCSQ), including active and negative coping score, and the coping tendency score[^5^](#_ENREF_5). For detail, the SSRS contains three subscales of social support: subjective support, objective support and the utility of support. Subjective support reflects the perceived interpersonal network that a person could bank on (4 items with scores ranging from 8-32). Objective support refers to the actual support a person gained in the past (3 items with scores ranging from 1-22). The utility of support reflects personal active seeking of various social supports (3 items with scores ranging from 3-12). The total SSRS scores are the sum of all three subscales, and higher scores (ranging from 12-66) imply stronger social support. The SCSQ contains assessments of active (12 items) and negative coping (8 items), respectively. The scale of each item uses 4-level Likert score standards, in which ‘3’ stands for regular use, while ‘0’ stands for no use. Then the scores for active and negative coping are measured independently, and a higher score indicates the inclination to adopt the corresponding coping style, while the coping tendency scores are defined as the active coping scores minus the negative coping scores.

1. **Supplementary Tables**

**Supplementary Table 1.** Primer sequences for four *FKBP5* SNPs genotyped in this study

| **SNP** | **Chromosome position** | **PCR Primer** | |
| --- | --- | --- | --- |
| rs3800373 | 35542476^a^ | Forward: | GAAAAGCGAGCAACTGCGTGTC |
|  |  | Reverse: | GGATCCATGCAGCTTTTTTTGTC |
| rs9296158 | 35567082^a^ | Forward: | CTCATTCCATGCCCAATAAAACAA |
|  |  | Reverse: | AGGCCTGGGCTAGGGGTAATTC |
| rs1360780 | 35607571^a^ | Forward: | ATGCTGAGGACAGCCTGCAAAG |
|  |  | Reverse: | TTAATATCTCTTGTGCCAGCAGTAGCA |
| rs9470080 | 35646435^a^ | Forward: | GGCTATGAATTGACAAAAAGCAGCTAA |
|  |  | Reverse: | tgtgtccagccATGTGCTTTTTTA |

^a^ Reference Genome: hg19

SNP = single nucleotide polymorphism; PCR = Polymerase chain reaction.

**Supplementary Table 2.** Alleles for four *FKBP5* SNPs genotyped in this study

| **SNP** | **Chromosome position^a^** | **Gene^a^** | **Alleles 1-2** | **11** | **12** | **22** |
| --- | --- | --- | --- | --- | --- | --- |
| rs3800373 | 35542476 | 3’ - UTR | C-A (0.3048) | 0.10 | 0.41 | 0.49 |
| rs9296158 | 35567082 | Intron 6 | A-G (0.3548) | 0.14 | 0.43 | 0.43 |
| rs1360780 | 35607571 | Intron 3 | T-C (0.2952) | 0.10 | 0.40 | 0.50 |
| rs9470080 | 35646435 | Intron 2 | T-C (0.3548) | 0.14 | 0.42 | 0.43 |

^a^ Reference Genome: hg19

SNP = single nucleotide polymorphism. UTR = untranslated regions

| Protocols | Adults with PTSD (N=49) | | | | Adults without PTSD (N=130) | | | |
| --- | --- | --- | --- | --- | --- | --- | --- | --- |
|  | H1/H1 (N=23) | H1/H2 (N=21) | H2/H2 (N=5) | *P* value | H1/H1 (N=64) | H1/H2 (N=51) | H2/H2 (N=15) | *P* value |
| Age (±SD), y | 58.04±5.87 | 57.38±5.53 | 58.00±5.57 | 0.92^a^ | 58.64±5.60 | 59.65±4.59 | 56.53±7.09 | 0.15^a^ |
| Sex (F/M) | 14/9 | 17/4 | 4/1 | 0.31^b^ | 26/38 | 26/25 | 8/7 | 0.46^b^ |
| Education, y | 6.65±3.94 | 5.86±4.50 | 7.80±5.02 | 0.63^a^ | 7.03±3.30 | 6.00±3.84 | 6.60±3.79 | 0.31 ^a^ |
| HAMD | 15.56±6.78 | 15.86±7.09 | 17.00±6.56 | 0.92^a^ | 5.56±4.14 | 6.12±3.91 | 6.87±5.25 | 0.51^a^ |
| HAMA | 12.34±6.88 | 12.95±7.24 | 12.80±4.15 | 0.96^a^ | 3.75±2.78 | 5.28±3.64 | 5.67±4.30 | 0.02^a^ |
| MMSE | 25.39±2.74 | 25.57±3.62 | 27.60±2.88 | 0.37^a^ | 26.43±3.98 | 25.59±2.89 | 26.67±2.50 | 0.35^a^ |
| Duration since trauma,  month | 49.43±38.47 | 63.71±62.29 | 38.14±17.06 | 0.54^a^ | 104.34±73.94 | 111.90±73.78 | 102.47±64.12 | 0.83^a^ |
| CAPS_total | 48.43±15.50 | 45.95±9.04 | 41.80±11.99 | 0.54^a^ | 16.03±9.84 | 17.08±10.32 | 15.27±9.68 | 0.78^a^ |
| **SSRS** |  |  |  |  |  |  |  |  |
| Objective support | 11.83±3.03 | 12.52±2.50 | 13.20±2.68 | 0.52^a^ | 12.82±2.66 | 12.64±2.84 | 11.71±2.81 | 0.40^a^ |
| Subjective support | 21.17±4.36 | 22.24±2.90 | 20.40±4.88 | 0.52^a^ | 21.89±3.88 | 21.18±3.79 | 20.57±4.32 | 0.41^a^ |
| Utility of support | 5.52±1.97 | 5.67±2.29 | 6.00±1.87 | 0.90^a^ | 5.33±1.92 | 5.57±1.87 | 5.86±2.32 | 0.60^a^ |
| SSRS_total | 38.52±7.68 | 40.43±6.28 | 39.60±8.88 | 0.68^a^ | 40.05±6.38 | 39.39±6.87 | 38.14±7.18 | 0.61^a^ |
| **SCSQ** |  |  |  |  |  |  |  |  |
| Active | 18.30±5.37 | 17.29±6.91 | 22.20±7.85 | 0.30^a^ | 19.79±6.50 | 18.53±6.69 | 20.57±5.37 | 0.45^a^ |
| Negative | 9.34±3.19 | 10.71±2.83 | 10.840±3.03 | 0.29^a^ | 10.03±3.28 | 10.84±3.59 | 10.21±2.64 | 0.43^a^ |
| Copying tendency | 8.96±4.87 | 6.58±5.59 | 11.40±8.56 | 0.16^a^ | 9.86±5.91 | 7.69±5.94 | 10.36±6.25 | 0.11^a^ |

**Supplementary Table 3: Demographics and clinical data of heterozygous/homozygous combinations of *FKBP5* H1, H2 haplotypes in Han Chinese adults who lost their only child**

Values are expressed as mean ± SD. PTSD = post-traumatic stress disorder; HAMD = Hamilton Depression; HAMA = Hamilton Anxiety; MMSE = Mini-Mental State Examination; CAPS = clinician-administered PTSD scale; SSRS = social support rating scale; SCSQ = simple coping style questionnaire.

^a^ The *P* value for the difference among the four genogroups was obtained by one-way analysis of variance test.

^b^ The *P* value for gender distribution among the four genogroups was obtained by the chi-square test.

**Supplementary Table 4: The effects of PTSD diagnosis, *FKBP5* diplotypes on spectral power in Han Chinese adults who lost their only child.**

| Brain regions | Brodmann  Area | MNI Coordinates | *F* value | Voxel number |
| --- | --- | --- | --- | --- |
| **Group main effect** |  |  |  |  |
| Parietal lobule, left (slow-4) | 7/19/40 | -30,-63,48 | 24.50 | 187 |
| Parietal lobule, right (slow-4) | 7/40 | 33,-57,48 | 17.75 | 49 |
| IFG, left (slow-5) | 10 | -45,39,0 | 23.80 | 52 |
| **FKBP5 diplotypes main effect** |  |  |  |  |
| ACC, bilateral (slow-4) | 33 | -6,9,27 | 10.73 | 54 |
| MCC, right (slow-3) | 31/24 | 9,-27,39 | 14.68 | 104 |
| Precentral/postcentral gyrus,  left (slow-3) | 3/4 | -39,-18,42 | 10.43 | 49 |
| Precentral/postcentral gyrus,  right (slow-3) | 3/4 | 48,-18,45 | 23.16 | 185 |
| **Groups × FKBP5 diplotypes**  **interaction effect** |  |  |  |  |
| Parietal lobule, right (slow-3) | 40 | 30,-42,-42 | 10.09 | 50 |

PTSD = post-traumatic stress disorder; BA = Brodmann area; IFG = inferior frontal gyrus; ACC = anterior cingulate cortex; MCC = middle cingulate cortex.

1. **Supplementary Figures**

**
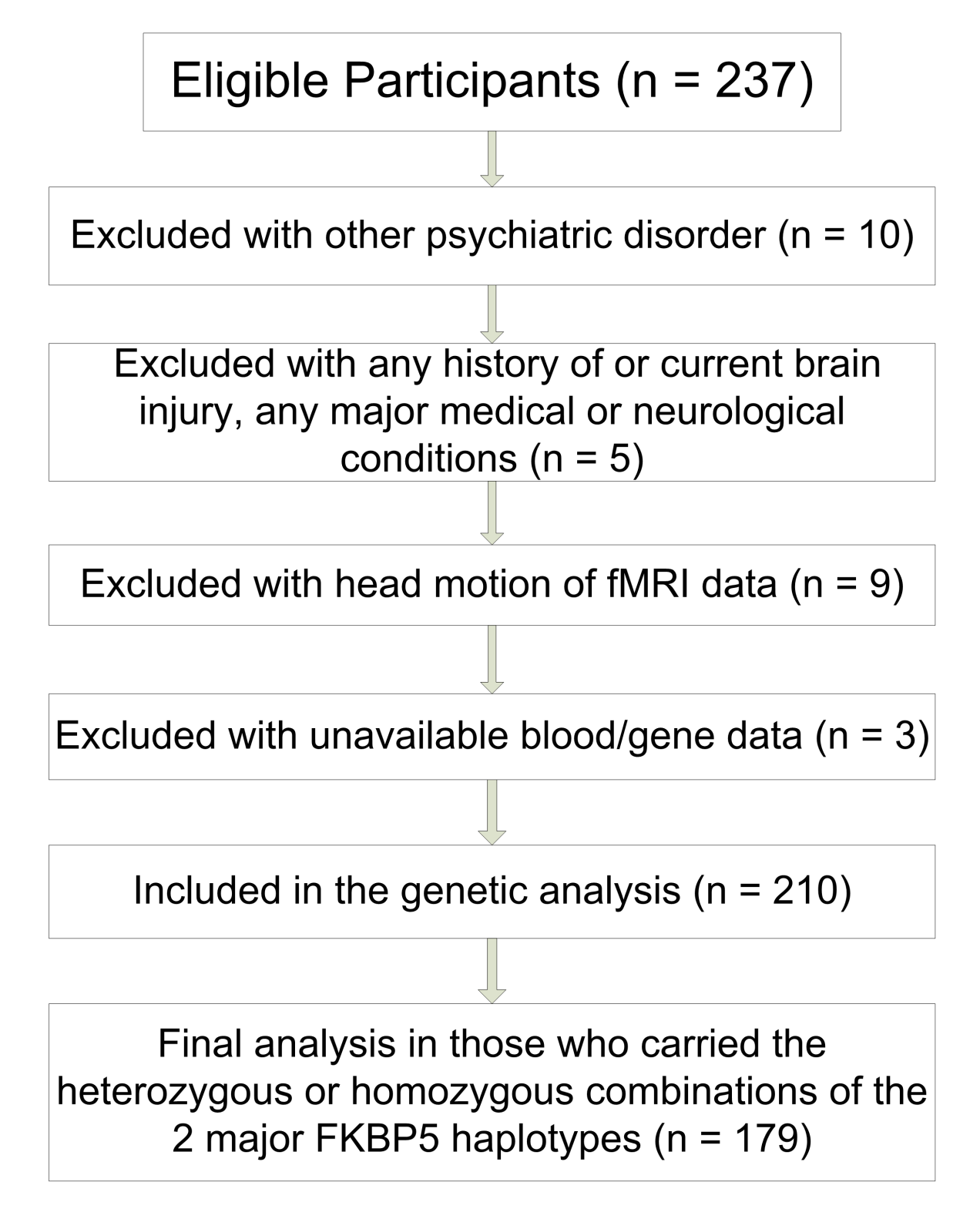
**

**Supplementary Figure 1.** The flowchart of the study population in this study.


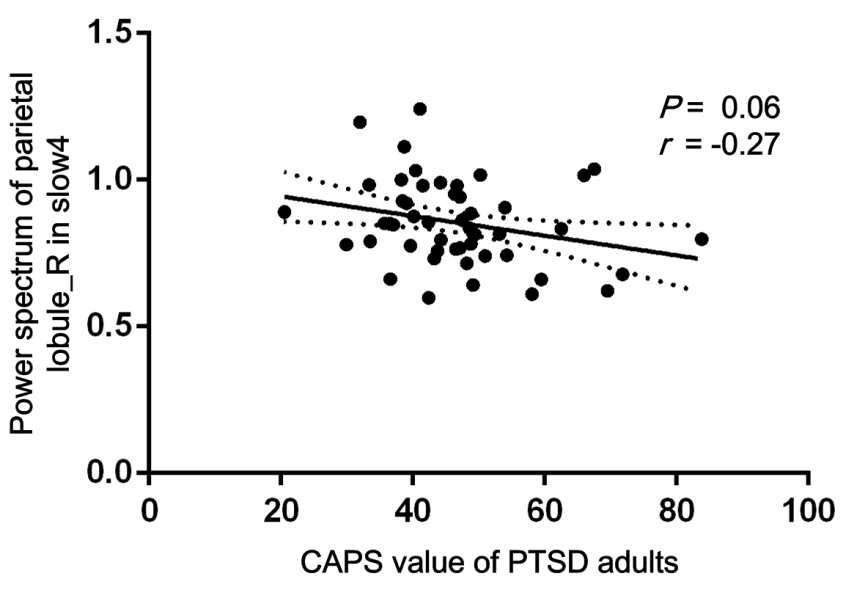


**Supplementary Figure 2.** Partial correlation results between the power spectrum in parietal lobule and CAPS scores (uncorrected *P*).

There is a marginally negative partial correlation between the power spectrum in parietal lobule and CAPS scores, only in the PTSD group (*r* = -0.27; *P* = 0.06)

PTSD = post-traumatic stress disorder. CAPS = clinician-administered PTSD scale

**REFERENCES**

1. Hamilton M. A rating scale for depression. *J Neurol Neurosurg Psychiatry* 1960; **23**(1)**:** 56.

2. Hamilton M. The assessment of anxiety states by rating. *Br J Med Psychol* 1959; **32**(1)**:** 50-55.

3. Folstein MF, Robins LN, Helzer JE. The Mini-Mental State Examination. *Arch Gen Psychiatry* 1983; **40**(7)**:** 812.

4. Cheng Y*, et al*. Social support plays a role in depression in Parkinson's disease: a cross-section study in a Chinese cohort. *Parkinsonism Relat Disord* 2008; **14**(1)**:** 43-45.

5. Jiang X-R, Du J-J, Dong R-Y. Coping style, job burnout and mental health of university teachers of the millennial generation. *EURASIA J Math, Sci Tech Ed* 2017; **13**(7)**:** 3379-3392.
